# Hypomagnesemia in critically ill patients

**DOI:** 10.1186/s40560-018-0291-y

**Published:** 2018-03-27

**Authors:** Bent-Are Hansen, Øyvind Bruserud

**Affiliations:** 1 Department of Microbiology, Førde Hospital, Førde, Norway; 2 Section for Endocrinology, Department of Clinical Science, University of Bergen, Bergen, Norway

**Keywords:** Magnesium, Critical illness, Intensive care unit, Arrhythmia, Potassium, Calcium

## Abstract

**Background:**

Magnesium (Mg) is essential for life and plays a crucial role in several biochemical and physiological processes in the human body. Hypomagnesemia is common in all hospitalized patients, especially in critically ill patients with coexisting electrolyte abnormalities. Hypomagnesemia may cause severe and potential fatal complications if not timely diagnosed and properly treated, and associate with increased mortality.

**Main body:**

Mg deficiency in critically ill patients is mainly caused by gastrointestinal and/or renal disorders and may lead to secondary hypokalemia and hypocalcemia, and severe neuromuscular and cardiovascular clinical manifestations. Because of the physical distribution of Mg, there are no readily or easy methods to assess Mg status. However, serum Mg and the Mg tolerance test are most widely used. There are limited studies to guide intermittent therapy of Mg deficiency in critically ill patients, but some empirical guidelines exist. Further clinical trials and critical evaluation of empiric Mg replacement strategies is needed.

**Conclusion:**

Patients at risk of Mg deficiency, with typical biochemical findings or clinical symptoms of hypomagnesemia, should be considered for treatment even with serum Mg within the normal range.

## Background

Magnesium (Mg) is essential for life and plays a crucial role in several biochemical and physiological processes in the human body. Hypomagnesemia is common in hospitalized patients (7–11%) and even more frequent in patients with other coexisting electrolyte abnormalities [1–3] and in critically ill patients [4, 5]. Hypomagnesemia can potentially cause fatal complications including ventricular arrhythmia, coronary artery spasm, and sudden death. It also associates with increased mortality and prolonged hospitalization [6, 7]. The role of Mg status and therapy in critically ill patients has previously been systematically reviewed elsewhere [8–10]. However, we here present a review article focusing on the Mg homeostasis and the physiological role of Mg in humans. We then present the different causes and clinical and biochemical manifestations of hypomagnesemia in critically ill patients and, finally, we discuss Mg therapy in the intensive care unit (ICU) setting.

## Magnesium homeostasis

**Fig. 1:**
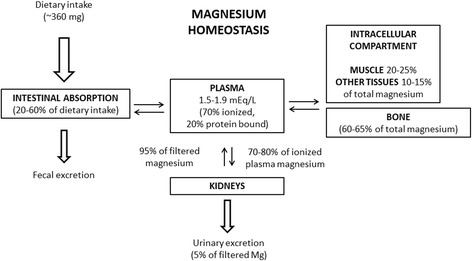
Mg homeostasis. The figure gives an overview of the Mg homeostasis and the distribution of Mg throughout the human body including gastrointestinal absorption and renal excretion

Mg is the fourth most abundant cation in the human body and the second most abundant intracellular cation. A healthy human adult have a content of about 25 g or 1000 mmol Mg where approximately 60% stores in bones, 20% in muscles, 20% in soft tissues, 0.5% in erythrocytes, and 0.3% in serum [11]. About 70% of the plasma Mg is ionized or complexed to filterable ions, while 20% is bound to proteins. Figure 1 gives a general overview of the Mg homeostasis in the human body.

Mg homeostasis in humans mainly involves the kidneys, the small bowel, and bones [12]. Gastrointestinal absorption and renal excretion are the most important mechanisms for controlling and regulating the Mg homeostasis. The cellular regulation of Mg uptake and release occurs slowly, and healthy individuals need to ingest about 0.15–0.2 mmol/kg/day to contain a normal Mg status. The intestinal absorption of dietary Mg depends on both intake and body Mg status and occurs via passive and active pathways [13, 14]. Presumably, only ionized Mg is absorbed. Active transcellular Mg uptake rely on specific Mg channels located in the large intestines [14, 15] including the transient receptor potential melastin (TRPM) 6 and TRPM 7. The passive absorption is driven by a favorable electrochemical gradient and occurs mainly paracellulary through leaky epithelia primarily located in the small intestines [14]. Additionally, the process of passive absorption interacts with the levels and absorption of calcium [14].

**Fig. 2:**
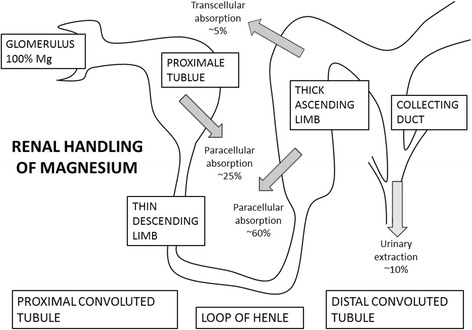
Renal Mg handling. The figure gives an overview of the renal handling of Mg in the proximal convoluted tubule, loop of Henle, and distal convoluted tubule

The kidneys are the primary site of Mg homeostasis and play a key role in regulating and maintaining Mg balance. Figure 2 illustrates the renal handling of Mg in humans. The normal fractional urinary excretion of filtered Mg is about 5% [16]. Mg reabsorption in the kidneys involves the proximal tubule, the thick ascending loop of Henle (TAL), and the distal tubule [17, 18]. TAL is the major site of Mg reabsorption and reabsorbs about 60–70% of filtered Mg [17, 18], and extracellular calcium sensing receptors modulate the Mg absorption through changes in the transepithelial voltage and alterations of the permeability of the paracellular tight junctions [18]. The mechanisms of basolateral transport into the interstitium are not fully understood. Moreover, the proximal tubule reabsorbs 15–20% of filtered Mg, and the distal tubule only 5–10% [17, 18], whereas there is no significant reabsorption of Mg in the collecting ducts [19].

## Physiological role of magnesium

Mg is a crucial cofactor in several enzyme systems [20] including almost every aspect of biochemical metabolism (e.g., DNA and protein synthesis, glycolysis, oxidative phosphorylation). The essential enzymes adenylate cyclase and sodium-potassium-adenosine triphosphatase depend on Mg for their normal function [21, 22]. Mg serves as a molecular stabilizer for RNA, DNA, and ribosomes. It is also suggested to modulate immune functions [23, 24], and changes in the level of Mg are reported to correlate with the levels of several immune mediators such as interleukin-1, tumor necrosis factor-alpha, interferon-gamma, and substance P [25–27]. Moreover, Mg are proven to contribute in several physiological processes such as maintaining stability across cell membranes, protein, and nucleic acid synthesis; regulating cardiac and smooth muscle tone; controlling of mitochondrial functions; and supporting cytoskeletal integrity [28].

## Defining magnesium status

Normal serum concentration of Mg is 1.5 to 1.9 mEq/L [29]. Unfortunately, there are no readily and easy methods to assess Mg status. However, serum Mg and the Mg tolerance test are most widely used [30]. The serum Mg is easily available but may not adequately reflect the body Mg stores because of the physiological distribution of Mg. Notably, normal serum levels may be found even if a patient is intracellularly Mg depleted because intracellular stores are recruited to keep the serum levels within its range, however, only until the point where these stores cannot keep up. Although only free Mg is biologically active, most test measure total Mg concentrations, and hypoalbuminemic states may therefore lead to false low Mg levels.

The Mg tolerance test is probably the most accurate way to assess Mg status [31]. The test is used in special occasions for example if the clinical suspicion of Mg deficiency is strong and the serum Mg levels are normal. The test is performed by measuring the Mg in a 24-h urine collection, distribute parenteral Mg (often 2.4 mg/kg of lean body weight given over the initial 4 h of the second urine collection), and then repeat the 24-h urine collection. Patients with a normal Mg status will excrete the Mg load during the second urine collection. Retention of more than 20% of the administrated Mg is suggestive of deficiency. Performing the test both gives the diagnosis and treats a potential Mg deficiency. The Mg tolerance test could easily be implemented in ICU patients. However, patients with malnutrition, cirrhosis, diarrhea, or long-term diuretic use typically have a positive result and the test is not useful in the setting of renal Mg wasting or other renal dysfunctions.

An alternative to the total serum Mg and the Mg tolerance test is assessment of the ionized serum Mg^2+^ concentration which is the active form of Mg in plasma [32]. It has a significant protein bound fraction, similar to calcium, with the potential of large differences between total serum and ionized levels [33]. The estimation of ionized Mg levels in patients cannot be made by correcting for albumin [33]. Notably, it is still disputable whether levels of serum ionized Mg or total serum Mg should be used to follow-up Mg levels in critically ill patients [7, 33–40]. Finally, the intracellular levels of Mg can be measured using circulating red blood cells, mononuclear cells, or skeletal muscle cells. Due to the lack of an accurate and robust method to measure Mg status in patients, the biochemical measurements should always be supported by a clinical assessment of patients at risk for Mg deficiency for timely and proper diagnosis and treatment.

## Causes of hypomagnesemia in critically ill patients

The causes of hypomagnesemia in critically ill patients are mainly a result of gastrointestinal disorders or renal loss of Mg. Table 1 lists the differential diagnosis of Mg deficiency in the ICU patients.

**Table 1:** Differential diagnosis of Mg deficiency in the ICU setting

Gastrointestinal disorders
Prolonged nasogastric suction
Malabsorption syndromes
Extensive bowel resection
Acute and chronic diarrhea
Intestinal and biliary fistulae
Protein-calorie malnutrition (parenteral nutrition, anorexia, refeeding syndrome)
Acute hemorrhagic pancreatitis
Primary intestinal hypomagnesemia (neonatal)
Renal loss
Chronic parenteral fluid therapy
Osmotic diuresis (glucose, mannitol, urea)
Hypercalcemia
Alcohol
Drugs (see Table 2)
Metabolic acidosis (starvation, ketoacidosis, alcoholism)
Renal diseases
Chronic pyelonephritis, interstitial nephritis, and glomerulonephritis
Diuretic phase of acute tubular necrosis
Postobstructive nephropathy
Renal tubular acidosis
Post-renal transplantation
Primary renal hypomagnesemia

### Gastrointestinal causes

Both the upper and lower intestinal tract fluid contain Mg. Therefore, loss of gastrointestinal fluids can cause Mg deficiency. Several conditions commonly seen in the ICU patients can cause gastrointestinal loss of Mg leading to significant Mg depletion, such as vomiting and nasogastric suction, diarrhea, enteritis, inflammatory bowel disease, intestinal and biliary fistulas, intestinal surgery resections, and pancreatitis [41–44].

### Renal causes

Many critically ill patients have hypomagnesemia caused by renal loss. The reabsorption of Mg^2+^ in the proximal tubule is proportional to tubular fluid flow and sodium reabsorption [17], and chronic parenteral fluid therapy, particularly with sodium-containing fluid, may therefore lead to Mg deficiency. The same mechanism may cause urinary wasting of Mg in osmotic diuresis. However, the most frequent cause of renal Mg wasting is medications, diuretics being particularly important [45]. Carbonic anhydrase inhibitors, osmotic agents, furosemide, bumetanide, and ethacrynic acid all increase Mg excretion [46], whereas the effect of thiazide diuretics on renal Mg handling is controversial [45]. Moreover, the aminoglycoside antibiotics [47], the chemotherapeutic agent cisplatin [48], and the immunosuppressive agent cyclosporine [49] are all reported to cause renal Mg wasting potentially causing Mg deficiency. Notably, patients in the ICU often receive several different combinations of intravenous medications and might have impaired drug elimination capacity due to reduced kidney and/or lived function which together with potential drug-drug interactions might influence Mg homeostasis. This aspect should be considered by physicians treating ICU patients. Table 2 gives an overview of the drugs that potentially cause hypomagnesemia and their underlying mechanisms. Finally, metabolic acidosis due to diabetic ketoacidosis, starvation, or alcoholism also causes renal Mg wasting.

**Table 2:** Drugs associated with Mg deficiency and hypomagnesemia

Drugs	Mechanisms causing Mg deficiency	Ref
	Renal loss	
Diuretics		
Loop	Increased renal Mg excretion by affecting the transepithelial voltage and inhibiting passive absorption.	[118]
Thiazides	Enhance Mg entry into the cells in the distal convoluted tubule.	[118]
Antimicrobial		
Amphotericin B Aminoglycosides Capreomycin Pentamidine	Renal urinary Mg wasting caused by nephrotoxins may be part of tubular necrosis and acute renal failure. Notably, impairment in Mg reabsorption in the loop of Henle and distal tubules may occur before the onset and may persist after the resolution of renal damage.	[19, 47, 119]
Chemotherapy		
Cisplatin	Renal urinary Mg wasting caused by nephrotoxins may be part of tubular necrosis and acute renal failure. Cisplatin treatment is also associated with lowered intestinal absorption	[120]
Immunosuppressive		
Calcineurin inhibitors	Urinary Mg wasting due to a downregulation of the Mg^2+^ transport proteins (TRPM6) in the loop of Henle and distal convoluted tubules.	[121]
Epidermal growth factor receptor inhibitors		
Cetuximab Panitumumab Matuzumab	Urinary Mg wasting due to a downregulation of the TRPM6 in the loop of Henle and distal convoluted tubules.	[122, 123]
	Gastrointestinal loss	
Proton-pump inhibitor	Impairing the intestinal Mg absorption by inhibiting Mg transporters (TRPM6 and TRPM7).	[124, 125]
	Miscellaneous	
Foscarnet	A general potent chelator of divalent cations which therefore has the potential to reduce ionized levels of Mg.	[126]
Cardiac glycosides	Mg deficiency is associated with cardiac glycosides. The exact mechanisms are not known.	[65]

## Biochemical and clinical manifestations of hypomagnesemia

Hypomagnesaemia is often secondary to other disease processes or drugs and the features of the primary disease may mask the signs of an Mg deficiency. Thus, a high index of suspicion is warranted [50]. An overview of the biochemical and clinical manifestations of hypomagnesemia are given in Table 3.

**Table 3:** Clinical and biochemical effects of moderate to severe Mg deficiency and hypomagnesemia

Biochemical
Hypokalemia
Renal K wasting
Decreased intracellular K
Hypocalcemia
Impaired parathyroid hormone secretion
Renal and skeletal resistance to parathyroid hormone
Resistance to vitamin D
Neuromuscular
Tetany
Spontaneous carpal-pedal spasm
Seizures
Vertigo, ataxia, nystagmus, athetoid, and choreiform movements
Muscular weakness, tremor, fasciculation, and wasting
Psychiatric: depression, psychosis
Cardiovascular
Dysrhythmias
Ventricular tachycardia (torsade de pointes)
Atrial fibrillation
Supraventricular tachycardia
Hypertension
Vasospasm
Electrocardiographic changes
Prolonged QT interval
Prolonged PR interval
Wide QRS
Peaked T waves
ST depression
Others
Acute myocardial infarction
Acute cerebral ischemia
Asthma exacerbation
Preeclampsia

### Biochemical manifestations of hypomagnesemia

#### Hypokalemia

Hypokalemia is common in patients with Mg deficiency and about half of the patients with clinically potassium deficiency also have Mg depletion [51]. However, patients with Mg depletion have a renal loss of potassium which is caused by an increased potassium secretion in the connecting tubule and the cortical collecting tubule. In the kidneys, K^+^ is absorbed across the basolateral membrane via Na-K-ATPase and secreted into the lumen of the connecting tubule and cortical collecting tubule. This process is mediated by luminal potassium channels (ROMK). With a total lack of intracellular Mg^2+^, K^+^ ions move freely through the ROMK channels. At physiologic intracellular Mg^2+^ concentration, ROMK conducts more K^+^ ions inward than outward. Hypomagnesaemia is associated with reduction of intracellular Mg, which in turn will release this inhibitory effect on potassium efflux. Due to the high concentration of potassium in the cell, this will promote potassium from the cell into the lumen which in turn leads to increased loss of potassium in the urine [52].

#### Hypocalcemia

Hypocalcemia is a well-known manifestation of Mg deficiency [53]. Patients with combined hypocalcemia and hypomagnesemia also show low levels of parathyroid hormone (PTH), and studies indicate that Mg deficiency inhibit the release of parathyroid hormone (PTH) in patients with coexisting hypocalcemia. Moreover, parenteral Mg stimulate PTH secretion [54, 55], and it is therefore suggested that reduced PTH secretion is a key contributor to hypocalcemia in Mg deficiency [55]. Animal studies have suggested that bone resistance to PTH contributes in hypocalcemia in Mg deficiency and studies in isolated perfused bone have revealed that Mg depletion reduces production of cyclic adenosine monophosphate (AMP) in bone with high levels of PTH [56]. Patients with Mg deficiency and hypocalcemia also present low levels of calcitriol (1.25-dihydroxyvitamin D) and together with impaired PTH secretion a reduced conversion of 25-hydroxyvitamin D to 1.25-dihydroxyvitamin D in the kidneys is suspected [57].

### Clinical manifestations of hypomagnesemia

#### Cardiovascular

Mg has several effects on the cardiac conduction system. It is an essential cofactor of the Na-K-ATP pump which controls the movement of sodium and potassium across cell membranes [58]; Mg levels therefore influence myocardial excitability. Typical electrocardiogram changes and dysrhythmias are most common [59]. Widening of the QRS complex and peaking of T waves are described in moderate Mg deficiency whereas prolongation of the PR interval, progressive widening of the QRS complex, and diminution of the T wave are seen in severe Mg depletion [19]. Low serum Mg has been correlated to increased risk of atrial fibrillation (AF) after cardiac surgery, and also an association between serum Mg and development of AF in individuals without cardiovascular disease is described [59]. Ventricular premature complexes, polymorphic ventricular tachycardia, and ventricular fibrillation are more severe complications [60, 61], and these arrhythmias may be resistant to treatment [62]. Intracellular Mg depletion may be present even with normal serum Mg levels and must always be considered as a potential factor in arrhythmias. Other electrolyte disturbances such as potassium or calcium deficiency are often concurrent but not obligate [63, 64]. Notably, both cardiac glycosides such as digitalis and Mg deficiency inhibit Na-K-ATPase and their adaptive effect contributes to increased toxicity [65].

Patients with heart failure have an increased incidence of hypomagnesemia probably due to the use of diuretics (Table 2). Non-potassium-sparing diuretics reduce serum and total-body potassium and Mg. Low levels of Mg and potassium predispose for ventricular ectopic activity which is a predictor for arrhythmic death [66]. However, there is conflicting evidence regarding Mg levels and cardiovascular death in patients with heart failure. A large prospective study did not find Mg depletion as an independent risk factor for death [67] whereas an association between low levels of serum Mg and cardiovascular mortality has been reported by others [68]. Mg supplementation has previously been suggested for patients with heart failure [69, 70].

Postoperative atrial fibrillation following coronary bypass (CAPG) occurs in 10–65% of the patients [71]. Hypomagnesemia is common after cardiac surgery and Mg levels drop significantly and remain decreased for about 24 h postoperatively [72–74]. The exact mechanisms are not known but may be due to hemodilution and renal wasting. Citrate in predeposited autologous blood may also contribute to the decrease in the serum Mg concentration [74]. Postoperative hypomagnesemia is associated with a higher incidence of postoperative arrhythmias and low cardiac index [73]. A meta-analysis of seven double-blinded, placebo-controlled, randomized clinical trials demonstrated that intravenous Mg significantly reduced the incidence of postoperative atrial fibrillation [75]. Notably, severe complications such as hypotension, progressive respiratory failure, diminished deep tendon reflexes, complete heart block, and cardiac arrest have been reported in overdosing of Mg [76].

Recent studies investigating Mg therapy in acute myocardial infarction (AMI) indicate that low serum Mg levels increases the frequency of arrhythmias [77] and that intravenous Mg supplements reduce the frequency of ventricular arrhythmias in AMI [78]. Three large prospective studies have investigated the role of Mg after AMI. “The Leicester Intravenous Mg Intervention Trial (LIMIT-2),” from 1992, randomized 2316 patients with suspected AMI to receive Mg or placebo and found a 24% relative reduction in 28-day mortality rate in patients receiving Mg therapy compared to the placebo group [79]. The Fourth International Study of Infarct Survival Trial (ISIS-4) randomized 58,050 participants in a 2 × 2 × 2 factorial study. The treatment comparisons were captopril vs placebo, mononitrate vs placebo, and intravenous Mg vs placebo. There was no significant reduction in 5 weeks mortality [80]. The MAGIC trial was published in 2002 with the purpose to investigate early administration of intravenous Mg to high-risk patients with acute myocardial infarction. Over 6000 patients with ST-elevation myocardial infarction (STEMI) were randomized to receive intravenous Mg or placebo and the study found no effect of early administration of intravenous Mg on 30-day mortality [81]. One of the main criticisms against ISIS-4 was the timing of the Mg administration as ISIS-4 randomized participants until 24 h after onset of symptoms whereas previously animal studies have stated that Mg must be given within 6 h after vessel occlusion [82, 83] for adequate effect. Moreover, Mg inhibits platelet activation by inhibiting Thromboxane A2 and interfering with the IIb-IIIa receptor complex [50], and Mg supplements are shown to inhibit platelet-dependent thrombosis in patients with coronary artery disease [84]. Based on the above, there are no indications of Mg in AMI as a routine but it may be considered in selected situations [85].

#### Neurovascular

Animal models suggest a cerebroprotective effect of Mg [86] and Mg therapy given within 6 h of cerebral infarction probably reduce tissue damage [87]. Regarding human studies, the intravenous Mg efficacy in stroke trial (IMAGES) randomized 2589 patients to receive intravenous Mg or placebo within 12 h of stroke onset but this did not reduce mortality or disability [88]. Moreover, a study investigating administration of prehospital Mg sulfate in acute stroke (FAST-MAG) did not find reduction in disability at 90 days after disease onset [86]. Neither has intravenous Mg been found to improve clinical outcome in aneurysmal subarachnoid hemorrhages [89].

#### Neuromuscular

Neuromuscular hyperexitability is often the first clinical manifestation in patients with hypomagnesemia [90]. Concomitant Mg and calcium deficiency enhance neurological symptoms, but also patients with isolated Mg deficiency present neuromuscular hyperexitability [50]*.* Other neuromuscular symptoms are tetanus with positive Chvostek and Trousseau signs, muscle spasms, and cramps [19] which probably all are due to lowering of the threshold for nerve stimulation [91]. Hypomagnesaemia may also affect neurons in the brain and cause seizures, likely due to increased glutamate-activated depolarization. A decrease of extracellular Mg^2+^ allows a greater influx of calcium in the presynaptic nerves and releases a greater amount of neurotransmitters [92, 93]. Choreiform and athetoid movements, vertigo, apathy, delirium, and vertical nystagmus are also described [94]. Vertical nystagmus is a rare but may be a diagnostically and useful sign of severe hypomagnesaemia. In absence of structural lesions in the cerebellum or vestibular device, vertical vertigo is only associated with severe hypomagnesaemia or thiamine deficiency [95].

#### Asthma

Mg is established treatment of resistant asthma attacks [96, 97]. Mg increases the effect of salbutamol [98] through inhibiting Ca^2+^ influx by blocking the voltage-dependent calcium channels which then relaxes the smooth muscle [28]. Mg also has an immunoregulatory effect by reducing pro-inflammatory mediators and promoting synthesis of prostacyclin and nitric oxide which stimulates broncho- and vasodilatation [99, 100]. Both intravenous and nebulized Mg has been used in treating acute asthma attacks. A review of 16 trials and 838 patients from 2012 showed that nebulized MgSO_4_ combined with a nebulized beta2 agonist in adults did not provide a benefit in terms of lung function or need for hospitalization [101]. Another Cochrane review included 25 trials with a total of 2907 patients. The aim was to determine efficacy and safety of inhaled MgSO_4_ administered in acute asthma due to lung function and hospital admission. The authors’ concluded with a modest additional benefit for the use of inhaled β_2_-agonists and ipratropium bromide [102].

Several systematic reviews and meta-analyses have assessed the role of intravenous or nebulized MgSO_4_ in acute asthma. A large double-blinded, placebo-controlled trial from 2013 included 1109 participants randomized to receive intravenous Mg, nebulized Mg, or placebo. The aim was to determine whether intravenous or nebulized MgSO_4_ improve symptoms of breathlessness and reduce the need for hospital admission in adults with severe acute asthma. The authors concluded that nebulized MgSO_4_ has no role in the management of severe acute asthma in adults and suggested a limited role for intravenous MgSO_4_ [103]. Another review including 2313 patients from 14 studies concluded that a single infusion of MgSO_4_ reduced hospital admissions and improved lung function in adults with acute asthma who did not respond sufficiently to standard treatments [104]. Interestingly, low Mg intake has been associated with a higher prevalence of asthma [105].

#### Preeclampsia

Mg therapy has been used for decades as eclampsia prophylaxis. In 2002, the results from the “Magnesium Sulphate for Prevention of Eclampsia trial” (MAGPIE) were published. Ten thousand patients with preeclampsia were randomized to receive Mg therapy or placebo. The Mg therapy group showed significant fewer cases of eclampsia compared to the placebo group, maternal death was fewer among women who received Mg therapy, and Mg did not seem to give harmful side effects to either the mother or the fetus [106]. There are conflicting evidence regarding the correlation between Mg depletion and preeclampsia [107–109]. However, based on MAGPIE, it seems reasonably using Mg therapy in patients with preeclampsia although the cellular mechanisms remain to be fully understood.

## Magnesium therapy

Because serum Mg not necessarily reflects the total body Mg status, patients at risk of magnesium deficiency or with symptoms consistent with hypomagnesaemia should be considered for treatment even with serum Mg within the normal range [19, 31]. The magnitude of Mg deficiency is hard to predict but may be 1–2 mEq/kg of body weight [50]. In general, mild hypomagnesemia with no or only mild symptoms can be treated with per oral supplement [110] whereas parenteral Mg supplementation is indicated if Mg concentration is < 0.5 mmol/L or if the patient presents with significant symptoms. For critically ill patients with mild to moderate hypomagnesemia, empirically derived “rules of thumb” suggest that the administration of 1 g (8 mEq) of intravenous Mg will increase the serum Mg concentration by 0.15 mEq/L within 18 to 30 h [111]. However, current practice of Mg replacement therapy is mainly based upon acute myocardial infarction trials (Table 4) which suggest an initial bolus (e.g., 2 g (16 mEq)) followed by continuous infusions up to 16 g (130 mEq) over 24 h [79, 112–114]. Severe hypomagnesemia may require treatment with doses until 1.5 mEq/kg; doses < 6 g MgSO_4_ can be given over a period of 8–12 h whereas higher doses should be administrated over a time period > 25 h [115]. The slow distribution of Mg in tissues and the rapidly renal excretion makes the infusion time crucial.

**Table 4:** Continues Mg infusions over 24 h

Author	*N*	Age, years	Male (%)	Serum creatinine (mg/dL)	Dose/diluent over 24 h	Serum change (mEq/L)	mEq/L rise/g Mg given
Shechter et al. [112]	96	66	65	≤ 3	130 mEq/500 mL 5% dextrose in water	1.65–2.82	0.007
Raghu et al. [113]	169	52.9	85	≤ 3	146 mEq/100 mL 0.9% NaCl	1.3–3.6	0.11
Rasmussen et al. [114]	56	64.6	70	≤ 3	100 mEq/1000 mL 5% dextrose in water	1.5–2.46	0.08
Woods et al. [79]	1159	61.4	74	≤ 3.4	146 mEq/50 mL 0.9% NaCl	1.64–3.1	0.08

Adapted from [127]

In the acute clinical settings with hemodynamically unstable patients, including patients with severe arrhythmias, established recommendations suggest giving 16 mEq (8 mmol) of Mg over 2–15 min followed by a continuous infusion [116, 117]. In the MAGPIE study [106], 32 mEq (4 g) Mg was initially given, followed by 8 mEq (1 g) per hour in women with preeclampsia [106]. Table 5 gives an overview of suggested Mg therapy in specific clinical setting. The evidence of using Mg as a routine in other critical conditions such as asthma or CAPG is still insufficient.

**Table 5:** Treatment with Mg in specific clinical settings

Diagnose	Suggested Mg doses	Comments	Ref
Hemodynamically stable patients with severe symptomatic hypomagnesemia	1–2 g [8–16 mEq] (4–8 mmol) MgSO4 given initially over 5–60 min followed by an infusion 4–8 g [32–64 mEq] (16–32 mmol) given slowly over 12–24 h.	–	[116, 128]
Torsades de pointes	2 g [16 mEq] (8 mmol) over 2–15 min followed by a continuous infusion.	The rate of Mg infusion depends on the clinical situation. Rapid infusion is associated with hypotension and asystole.	[116, 117]
Preeclampsia	4 g [32 mEq] (16 mmol) over 10–15 min followed by 1 g [8 mEq] (8 mmol) every following hours.	Evidence is conflicting and no consensus about the optimal Mg regimen exists. Suggested loading doses vary from 4 to 6 g (32–48 mEq; 16–24 mmol) and maintenance doses of 1–3 g (8–24 mEq; 4–12 mmol)/h.	[106]

Patients with renal failure are at risk of developing hypermagnesemia and Mg treatment is therefore generally not recommended for these patients. However, Mg therapy should be considered in patients with moderately reduced glomerular filtration rate and severe Mg deficiency. The dose of Mg must be adjusted and patients should be carefully monitored both biochemically and clinically. High levels of Mg (> 4–5 mmol/L) may give muscle weakness, reduced respiration, and in worst case cardiac arrest. In case of intolerable intoxication; intravenous calcium (100–200 mg over 5–10 min) should be administrated as it antagonizes the neuromuscular and cardiovascular effects of Mg [50, 115].

## Conclusion

- Mg deficiency is common in critically ill patients, may cause potentially fatal complications, and associates with increased mortality.
- Mg deficiency in critically ill patients is mainly caused by gastrointestinal and/or renal disorders and may lead to secondary hypokalemia and hypocalcemia, and severe neuromuscular and cardiovascular clinical manifestations.
- Because of the physical distribution of Mg, there are no readily or easy methods to assess Mg status. However, serum Mg and the Mg tolerance test are most widely used.
- Patients at risk of Mg deficiency, with typical biochemical findings or clinical symptoms of hypomagnesemia, should be considered for treatment even with serum Mg within the normal range.
- There are limited studies to guide intermittent therapy of Mg deficiency in critically ill patients but some empirical guidelines exist. Further clinical trials and critical evaluation of empiric Mg replacement strategies is needed.
